# Supplementary material for: Breathwork Interventions for Adults with Clinically Diagnosed Anxiety Disorders: A Scoping Review
Source: Brain Sci. 2023 Feb 2;13(2):256. doi: 10.3390/brainsci13020256 (PMC9954474; doi:10.3390/brainsci13020256)
Supplement: Supplementary file 1 [file brainsci-13-00256-s001.zip › brainsci-2184824-supplementary.docx]

**Table S1.** Study research.

| Database | Query | Items found |
| --- | --- | --- |
| PubMed | ((breathwork OR "breathing exercise" OR "breathing technique" OR "breathing practice" OR "breath regulation" OR pranayama OR "mindful breathing" OR "paced breathing" OR "controlled breathing" OR "slow breathing" OR "fast breathing" OR hyperventilation OR "deep breathing" OR "metronome breathing" OR "nasal breathing" OR "mouth breathing" OR "diaphragmatic breathing")) AND (("Anxiety"[MeSH] OR "Anxiety Disorders"[MeSH] OR "Stress Disorders" OR stress OR anxiety OR phobia OR phobic OR panic OR "stress disorder" OR agoraphobia))  *Filters applied: Classical Article, Clinical Study, Clinical Trial, Controlled Clinical Trial, Randomised Controlled Trial, Humans, English, Adult: 19+ years, Young Adult: 19-24 years, Adult: 19-44 years, Middle Aged + Aged: 45+ years, Middle Aged: 45-64 years, Aged: 65+ years, 80 and over: 80+ years.* | 235 |
| Embase | *#1*  (breathwork OR 'breathing exercise' OR 'breathing technique' OR 'breathing practice' OR 'breath regulation' OR pranayama OR 'mindful breathing' OR 'paced breathing' OR 'controlled breathing' OR 'slow breathing' OR 'fast breathing' OR hyperventilation OR 'deep breathing' OR 'metronome breathing' OR 'nasal breathing' OR 'mouth breathing' OR 'diaphragmatic breathing') AND ('anxiety'/exp OR 'anxiety disorders'/exp OR 'stress disorders' OR stress OR anxiety OR phobia OR phobic OR panic OR 'stress disorder' OR agoraphobia) AND [article]/lim AND [english]/lim AND ([adolescent]/lim OR [adult]/lim OR [young adult]/lim OR [middle aged]/lim OR [aged]/lim OR [very elderly]/lim)  *#1 AND ('clinical study'/de OR 'clinical trial'/de OR 'controlled clinical trial'/de OR 'randomised controlled trial'/de)* | 387 |
| Scopus | (((breathwork OR "breathing exercise" OR "breathing technique"  OR  "breathing practice"  OR  "breath regulation"  OR  pranayama  OR  "mindful breathing"  OR  "paced breathing"  OR  "controlled breathing"  OR  "slow breathing"  OR  "fast breathing"  OR  hyperventilation  OR  "deep breathing"  OR  "metronome breathing"  OR  "nasal breathing"  OR  "mouth breathing"  OR  "diaphragmatic breathing" )) AND  (( anxiety  OR  "Anxiety Disorders"  OR  "Stress Disorders"  OR  stress  OR  anxiety  OR  phobia  OR  phobic  OR  panic  OR  "stress disorder"  OR  agoraphobia)))  *Filters applied:*  AND  ( LIMIT-TO ( LANGUAGE ,  "English" ))  AND  ( LIMIT-TO ( SRCTYPE ,  "j" ) )  AND  ( LIMIT-TO ( DOCTYPE ,  "ar" ) )  AND  ( LIMIT-TO ( EXACTKEYWORD ,  "Adult" )  OR  LIMIT-TO ( EXACTKEYWORD ,  "Middle Aged" )  OR  LIMIT-TO ( EXACTKEYWORD ,  "Aged" )  OR  LIMIT-TO ( EXACTKEYWORD ,  "Young Adult" )  OR  LIMIT-TO ( EXACTKEYWORD ,  "Middle Age" )  OR  LIMIT-TO ( EXACTKEYWORD ,  "Aged, 80 And Over" ) )  AND  ( LIMIT-TO ( EXACTKEYWORD ,  "Randomised Controlled Trial" )  OR  LIMIT-TO ( EXACTKEYWORD ,  "Clinical Trial" )  OR  LIMIT-TO ( EXACTKEYWORD ,  "Controlled Clinical Trial" )) | 459 |
